# The Impact of Bilingualism on Everyday Executive Functions of English-Arabic Autistic Children: Through a Parent-Teacher Lens

**DOI:** 10.1007/s10803-021-05114-5

**Published:** 2021-06-06

**Authors:** Shereen Sharaan, Sarah E. MacPherson, Sue Fletcher-Watson

**Affiliations:** 1 Human Cognitive Neuroscience, Department of Psychology, The University of Edinburgh, Edinburgh, UK; 2 The Salvesen Mindroom Research Centre, University of Edinburgh, Edinburgh, UK

**Keywords:** Dual language, Second language exposure, Autism, Cognition

## Abstract

There is evidence that autistic children may have reduced executive function skills, contributing to day-to-day difficulties, but much remains unknown regarding the influence of bilingualism. We investigated its influence on sustained attention, interference control, flexible switching and working memory, in Arabic-English autistic (n = 27) and typically developing peers (n = 53) children, aged 5 to 12 years old. Parents and teachers completed rating measures assessing children’s daily EF abilities. Results showed generalized positive effects for bilingual autistic children relative to their monolingual peers across all EF domains, but using parent ratings only. The findings indicate that bilingualism does not negatively impact the executive function skills of autistic children, and that it might mitigate difficulties faced on a day-to-day basis.

**Supplementary Information:**

The online version contains supplementary material available at 10.1007/s10803-021-05114-5.

## Introduction

Given the rising diagnostic rates of autism and increases in the worldwide bilingual population, it is of interest to chart the effects of bilingualism in autism. The current investigation focuses on the influence of bilingualism on a set of executive function (EF) skills that have been identified as vulnerable in autism. All data were collected in the United Arab Emirates, a country that offers a predominantly dual-language switching environment (i.e., using two languages in the same context). According to the adaptive control hypothesis, a prominent theoretical account of the relationship between bilingualism and EF, this kind of linguistic environment provides the optimal context for bilingualism to have an impact on EF. To introduce this work, we first consider the interplay of EF and autism; then of EF and bilingualism in typical development; and finally, the intersection of autism, bilingualism and EF.

Executive functions (EFs) refer to a broad range of higher-order thinking skills that include, but are not limited to, sustained attention (the ability to focus over a period of time), interference control (the ability to resist distracting information), flexible switching (the ability to switch between thoughts and adapt behavior according to a changing environment), and working memory (the ability to simultaneously store and process information) (see McCloskey et al. (2009) for a review of EF definitions). In typically developing children, the role of executive functions has been well-established across a range of educational (Allan et al., 2014; Dekker et al., 2017) and social domains (Hughes et al., 2000; Murphy et al., 2004), as well as having an influence on quality of life (Tangney et al., 2004).

Autism is described as a lifelong neurodevelopmental spectrum condition with each individual varying in their abilities (strengths and difficulties) across social and cognitive domains*.* This combination of strengths and difficulties can vary over time. What characterizes autism is a complex collection of manifestations, namely, repetitive behaviors, social communication difficulties, and sensory hypersensitivity and hyposensitivity (American Psychiatric Association, 2013). Executive dysfunction in autism has been widely evidenced (Demetriou et al., 2017; Lai et al., 2017), though large individual differences in performance have also been demonstrated (Geurts et al., 2014; Pellicano, 2010). Deficits in executive function have been associated with low quality of life in autistic people (Vries & Geurts, 2015), and while it has not been evidenced to cause features of autism, it may be related to difficulties that characterize autism in social and non-social domains (Happé et al., 2006; Hill, 2004). Heterogeneity in autism has been clearly evidenced in the EF domain, however, our understanding of the elements that influence the development of EF and outcomes in autism remains poor (Demetriou et al., 2017). One factor that can influence EF development in autism could be exposure to two languages.

Bilingualism is an ability common to the majority of the world’s population, and broadly speaking, refers to the knowledge of more than one language (Grosjean, 2010). This includes oral and sign languages, and the knowledge of both is referred to as bimodal bilingualism. Bilingualism comprises an extensive spectrum of language experiences and markers. It encompasses individuals who: (a) are exposed to two languages from birth or a very early age (i.e., simultaneous bilinguals), (b) are exposed to the second language during childhood, after the first language is somewhat established (i.e., early sequential bilinguals), (c) are exposed to the second language after childhood (i.e., late sequential bilinguals), (d) possess equal abilities in their two languages (i.e., balanced bilinguals), (e) possess unequal abilities in their two languages (i.e., unbalanced bilinguals).

A spectrum of bilingualism markers has been associated with improved EF development in typically developing children, including language acquisition at an earlier age (Kapa & Colombo, 2013), higher proficiency in languages (Niharika & Ramesh Kumar, 2013), and regularly switching between languages (Prior & Gollan, 2011). The influence of bilingualism on EFs in typically developing children is a heavily debated topic, with findings in favor of (Barac et al., 2014; Bialystok, 2001) and against (Dick et al., 2019; Paap & Greenberg, 2013) a bilingual advantage (i.e., where bilingual participants significantly outperform monolingual participants on EF measures). For in-depth accounts of the complexities surrounding the literature on bilingualism and EF, see Paap and Greenberg (2013) and de Bruin et al. (2015). While there are null results (i.e., findings of equivalent EF performance between bilinguals and monolinguals), disadvantages in EF performance for bilingual typically developing (Dick et al., 2019) and bilingual autistic (Gonzalez-Barrero & Nadig, 2017) children are seldom in evidence.

One of the notable frameworks that propose an explanation for the relationship between bilingualism and EF is the Adaptive Control Hypothesis (ACH) (Green & Abutalebi, 2013). This framework argues that the relationship between bilingualism and EF is fundamentally determined by language context. In a ‘single language context’, the two languages are used in separate and distinct contexts (e.g., using English at work and Japanese at home), resulting in no switching between the two languages. In a ‘dual language context’, both language are used in the same context, resulting in regular switching between the two languages. Finally, in a ‘dense language context’, there is alternation between the two languages within single sentences, and/or adaptation of words from one language to fit another. The adaptive control hypothesis suggests that EF skills such as flexible switching, inhibitory control, and sustained attention would be most enhanced by a dual language context. This theoretical difference between language contexts may be one reason why evidence to date on the effect of bilingualism on EF is so equivocal.

The adaptive control hypothesis has received empirical support, particularly from studies investigating the EF domains of flexible switching and interference control in typically developing children. For instance, a study with early bilinguals highlighted that the frequency with which they switch between languages on a day-to-day basis significantly predicted error rates on an experimental flexible switching task (number-letter task) (Soveri et al., 2011). Another study using a color-shape flexible switching task demonstrated an advantage for early bilinguals who reported regular language switching, and equivalent performance between monolinguals and early bilinguals who reported less regular language switching (Prior & Gollan, 2011). Similarly, there is support for a link between interference control abilities (e.g., using a Flanker task) and frequency of language switching in early bilinguals (Verreyt et al., 2016). Together, these findings provide supporting evidence for the theoretical relationship between language switching and the prime abovementioned EF skills in bilinguals.

A limited but growing evidence-base shows that bilingualism does not result in disadvantages for autistic children in language domains (see Uljarević et al., 2016 for a review). In the domain of EF, the evidence is even more limited, but one study reported equivalent performance between autistic children who were exposed to a second language (i.e., exposure to any other spoken language in the home other than the primary language, as reported by parents) and those who were not, using parent ratings that assess problem solving, attentional control, behavioral control and emotional control (Iarocci et al., 2017). Similarly, equivalent performance between bilingual and monolingual autistic children was demonstrated for directly-assessed measures of inhibitory control and flexible switching (Li et al., 2017). In the study by Iarocci et al. (2017), the authors note that despite a lack of statistically significant differences in EF performance between autistic children with exposure to single or dual-language context, those with a second language exposure were less likely to present EF difficulties that are clinically concerning. These results were echoed in a more recent investigation by Ratto et al. (2020), where parents reported fewer EF difficulties in autistic participants who were on average four years old and exposed to a second language > 10% of the time, across inhibitory self-control and flexible switching outcomes.

Other studies provided preliminary support for the hypothesis that bilingual autistic children have an advantage compared to monolingual autistic children. A study by Li et al. (2017) revealed a bilingual advantage for age and NVIQ-matched autistic children who were eight years old on average and exposed to both languages before the age of three—on a directly-assessed outcome measure of inhibitory control. Another study by Gonzalez-Barrero and Nadig (2017) reported an advantage for autistic children (matched on age (6–9 years), NVIQ, maternal SES and gender) who had > 20% lifetime exposure to both languages and scored 3 or 4 on a 4-point scale of language proficiency in each language—on a directly-assessed outcome measure of flexible switching. More recently a study by Sharaan et al. (2021) published an advantage for autistic bilinguals (matched on age (5–12 years), NVIQ, maternal SES, and paternal SES) who had > 20% of current exposure to each of the two languages at home or school, > 20% of current active speaking in each of the two languages at home or school, and > 20% proficiency in each of the two languages—on a directly-assessed outcome measure of sustained attention, but not for other EF domains.

In each study, however, only one outcome variable (out of two or more outcome variables) revealed an advantage for autistic bilinguals, so it is not clear whether these findings are robust. Furthermore, in the second study (Gonzalez-Barrero & Nadig, 2017), no bilingual advantage was reported for autistic participants on a parent report measure assessing flexible switching. Taken together, these quantitative findings strongly support the idea that bilingualism is not harmful to autistic children’s language and EF development, and may result in some advantages.

The current investigation focuses on the influence of bilingualism on a set of EF skills that have been identified as vulnerable in autism and are relevant to the adaptive control hypothesis—sustained attention, flexible switching, and interference control. In addition, working memory is included as an active control EF but as it is not driven by the ACH model of interest, we hypothesize there will be no effects of bilingualism on working memory. We selected informant-report measures to capture “trait-like” everyday capacities, which are relatively stable over time (Samyn et al., 2015), seeking input from both teachers and parents to increase robustness of our findings. This investigation took place in a dual-language environment (the type of language context most likely to benefit our shortlisted EFs according to the ACH); the United Arab Emirates (UAE), where the presiding language of Arabic is in the top five most spoken languages of the world, with more than 240 million native speakers (Adams & Fleck, 2015).

In reference to the theoretical model under investigation (i.e., Adaptive Control Hypothesis), we hypothesize that bilingual children will outperform monolingual children on measures of flexible switching, sustained attention and interference control, on both parent and teacher ratings of EF. We hypothesize there will be no effects of bilingualism on working memory. In addition, we will explore interactions between diagnostic status and bilingualism in their effect on informant EF scores, but hold no firm hypothesis about EF performance between autistic and TD bilinguals. In relation to agreement between different informants, we will investigate whether parent and teacher ratings (a) correlate with one another, and (b) show differential patterns when comparing diagnostic (autism/TD) and language (bilingual/monolingual) groups.

## Methods

### Participants

One hundred and fifteen children aged between 5 and 12 years were recruited into the study (*M* = 111.37 months, SD = 21.43 months), but only 80 children had a consistency index standard score > 75 on the EF rating scale. The EF rating scale used (see ‘Materials’ section below for details) includes a consistency index—a metric of the internal consistency of participant responses. Inconsistent responding can take place in a deliberate or undeliberate manner, and may be attributed to deliberate resistance to follow instructions, tiredness, a misinterpretation of the items or instructions, distraction, indifference, or a lack of incentive. Therefore, the pattern of ratings is typical or consistent if the consistency index standard score > 75 (as stated in the manual of the measure). In contrast, the pattern of ratings is not typical or inconsistent if the consistency index standard score ≤ 75. By including data from participants with a consistent rating pattern only, we ensure reliable responses are being analyzed. Therefore, after excluding 35 participants whose raters showed an inconsistent response style, 80 participants with ratings showing a consistent response style were carried forward for analyses.

Monolingual children spoke either English (n = 47) or Arabic (n = 3), and all bilingual children spoke both English and Arabic (n = 30). All the children in this work had started schooling, either in mainstream or special education settings. All bilingual children spent considerable periods of time in dual-language switching contexts, where Arabic and English are utilized within the same context (i.e., school and/or home environments). For the majority of bilingual children, English was their dominant language. Typically developing bilingual children were all exposed to their second language by the age of 4 years, while the earliest age of exposure to a second language varied from 0 to 8 years for autistic bilingual children. According to Lenneberg’s theory (1967), early bilinguals start to use their L2 between the ages of 1–6 years. Therefore, while it is possible that some of our bilingual autistic children might be considered sequential language learners, most of the sample would be characterized as ‘early bilinguals’.

The primary inclusion criteria for autistic children was a formal clinical diagnosis of autism based on DSM-IV or DSM-V criteria (measures included either ADOS/ADOS-2 or ADI/ADI-R) obtained from licensed clinicians (i.e., psychologists) at educational or healthcare institutions. A copy of the diagnostic report to confirm participants’ diagnostic status was provided by a primary caregiver (i.e., a parent) or the educational institution where the participant is enrolled (i.e., schools and/or centers). Children with a range of non-verbal IQ scores on the Raven’s Colored Progressive Matrices (CPM; Raven et al., 1990) were recruited to increase inclusive participation into the study. The instrument has reportedly good reliability (reliability coefficient > 0.80) and validity (validity coefficient > 0.70) (Raven et al., 1990). Parents reported no intellectual, cognitive, visual or hearing impairments, as well as no co-morbidities (e.g., ADHD).

The Child Language Experience and Proficiency Questionnaire (Marian et al., 2007) (collecting language history, current exposure, current use and proficiency data) was administered to parents, in either Arabic or English. The instrument has reportedly good reliability (reliability coefficient > 0.70) (Marian et al., 2007). Expressive vocabulary was measured with the Picture Naming Test (PNT) (Kharkhurin, 2008) in Arabic and/or English. The English version has demonstrated high test–retest reliability (reliability coefficient > 0.80) while data on the Arabic version are lacking (Kharkhurin, 2008). Receptive vocabulary was measured with the Peabody Picture Vocabulary Test, 4th ed. (PPVT-4) (Dunn & Dunn, 2007). It is available in the English version, presenting strong psychometric properties for test–retest reliability; reliability coefficient > 0.90) (Dunn & Dunn, 2007). As an Arabic version does not exist, the Arabic Picture Vocabulary Test (APVT) was sourced to measure receptive vocabulary in Arabic. While this test is not standardized, it shows good correlations with the British Picture Vocabulary Test and has reportedly high internal reliability (reliability coefficient of 0.82) (Shaalan, 2010).

Children’s bilingual status was determined based on an amalgam of the following indices: (1) > 20% of current exposure to each of the two languages at home or school, according to the Child Language Experience and Proficiency Questionnaire (parent report); (2) > 20% of current active speaking in each of the two languages at home or school, according to the Child Language Experience and Proficiency Questionnaire (parent report); (3) > 20% proficiency score (20% is equivalent to 24 correct item-responses out of 120 items) in each of the two languages as per the Picture Naming Test (expressive vocabulary), scored by the researcher. Children’s monolingual status was determined based on a combination of the following indices: (1) had not been exposed to a language other than Arabic (or English if their first language was English) for more than 20% of their lifetime, according to the Child Language Experience and Proficiency Questionnaire (parent report); (2) if exposed to a second language, < 20% of current active speaking at home or school, according to the Child Language Experience and Proficiency Questionnaire (parent report); (3) if exposed to a second language, < 20% proficiency score (20% is equivalent to 24 correct item-responses out of 120 items) as per the Picture Naming Test (expressive vocabulary), scored by the researcher. Proficiency was determined based on the accuracy of children’s verbal responses to 120 items in the PNT. We note that 70% of our autistic bilingual children and 82% of our TD bilingual children have medium–high proficiency in both their languages, therefore, treating bilingualism as a continuous variable was not appropriate for our study.

Our adopted monolingual and bilingual thresholds are thus based on these study-specific parameters. Therefore, we do not consider bidialectalism (i.e., speaking a dialect in addition to a standard language; which is the case for some of our child participants) a form of bilingualism. Furthermore, the few studies that have examined bidialectism in relation to bilingualism have yielded mixed findings. Antoniou et al. (2016) found bidialects to perform between monolinguals and bilinguals on measures of EF. Kirk et al. (2014) and Ross & Milinger (2017) found bidialects to perform similarly to monolinguals on tasks of EF. Future studies that are specifically designed to disentangle the effects of bidialectism and bilingualism on EF are needed to shed further light on this. All monolingual children in this study, however are exposed to a second language in their community. This concern is ameliorated by some key factors nevertheless. Our monolingual children were below threshold on our robust proficiency criteria, drawn from monolingual homes and taught / instructed in one language at school, meaning that the impact of the wider cultural context was significantly diluted. Ultimately, our results should be interpreted as relevant to bilingual language use and proficiency, rather than mere passive exposure.

Ethical approvals were obtained from the University of Edinburgh (School of Philosophy, Psychology and Language Sciences, Application 102-1718/2), the Abu Dhabi Department of Education and Knowledge, and the UAE Ministry of Community Development. All parents and participants gave informed consent.

Details of participant characteristics are presented in Table 1. A series of one-way analysis of variance (ANOVA) confirmed significant differences between the 4 groups (autistic bilingual, autistic monolingual, TD bilingual, TD monolingual) on non-verbal IQ (*p* = 0.000). Post-hoc comparisons using the Tukey HSD test revealed that: (a) The TD bilingual group had significantly higher non-verbal IQ than the autistic monolingual group (*p* = 0.000), (b) The TD monolingual group had significantly higher non-verbal IQ than the autistic monolingual group (*p* = 0.000), and (c) The autistic bilingual group had significantly higher non-verbal IQ than the autistic monolingual group (*p* = 0.003). There were no significant differences between the groups on chronological age (*p* = 0.475), maternal continuous years of education (*p* = 0.568) or maternal minimum education level (*p* = 0.247).

**Table 1:** Participant Demographics by Group

	Monolingual		Bilingual		Range
	Autistic (N = 21) M (SD)	TD (N = 29) M (SD)	Autistic (N = 6) M (SD)	TD (N = 24) M (SD)	
Participant age (months)	104.76 (23.90)	114.48 (25.98)	116.17 (16.31)	110.08 (19.54)	59–153
CPM	6.76 (2.84)	2.52 (1.52)	3.50 (1.76)	2.42 (1.31)	1–9
Maternal education level	5.24 (1.64)	5.90 (1.29)	5.00 (1.89)	5.67 (0.86)	2–8
Maternal education (years)	16.10 (1.99)	16.62 (1.54)	16.83 (3.92)	16.83 (1.20)	12–24
Autistic symptomatology	69.80 (16.14)	–	67.91 (10.28)	–	53–91
Gender (M/F)	17/4	6/23	4/2	11/13	

*M* mean, *SD* standard deviation, *TD* typically developing, *CPM* Colored Progressive Matrices nonverbal IQ grade (Grade 1 = intellectually superior (score lies at or above the 95th percentile for individuals of that age group), *Grade 2, Grade 3* definitely above average (score lies at or above the 75th percentile for individuals of that age group), *Grade 4, Grade 5* intellectually average (score lies between the 25th and 75th percentile for individuals of that age group), *Grade 6, Grade 7* intellectually below average (score lies at or below the 25th percentile for individuals of that age group), *Grade 8* intellectually impaired (score lies at or below the 5th percentile for individuals of that age group). Autistic Symptomatology = as assessed by the Social Responsiveness Scale-2: 76 or higher (severe deficits), 66 to 75 (moderate deficits), 60 to 65 (mild to moderate deficits), 59 and below (no deficits).”

### Language Context

English is generally considered to be the lingua franca as approximately 90% of the UAE’s population is made up of non-citizens (De Bel-Air, 2015). In addition to the presence of an English-Arabic dual language environment, three versions of spoken Arabic are present, representing a triglossic context. These include: Classical Arabic (i.e., a version of Arabic adopted by the Quran and literary projects), Modern Standard Arabic (i.e., a version of Arabic used in formal communications, for example, schooling and the news media), and Colloquial Arabic (i.e., Arabic associated with dialects used in everyday-type contexts) (Sabbah, 2015). In a study focused on language education in the UAE, Al Sharhan (2007) stated that development of all three varieties of Arabic was a core aspect of Emirati children’s language education.

### Materials

The Comprehensive Executive Function Inventory (CEFI; Goldstein & Naglieri, 2014) is an EF rating scale comprised of 100 items for individuals aged 5–18 years, with both parent and teacher rating forms. The four sub-scales of the CEFI are: interference control, flexible switching, sustained attention, and working memory. Parents and teachers are asked to rate behaviors observed during the last four weeks. Standard scores < 90 indicate a weakness in executive function. The CEFI is highly correlated with similar and more widely used measures like the BRIEF (Gioia et al., 2000) but it is more precisely normed than the BRIEF (Goldstein & Naglieri, 2014) and also captures the ‘sustained attention’ EF domain, unlike the BRIEF. The test has reported very good to excellent internal reliability and test–retest stability (Goldstein & Naglieri, 2014). The CEFI is only available in English, and thus, an Arabic version was created with publisher approval—the procedure is detailed below.

For the initial translation, two independent forward translations were made from English to Arabic by bilingual translators whose mother tongue is Arabic. One of the translators did not have knowledge of the CEFI items being quantified (as per publisher requirements) nor a developmental or clinical background. A written report was produced based on each translation (T1 and T2) with comments regarding challenges and reasoning for their choices recorded. Both translations were then combined into one common translation (T3). Any challenges resulting from synthesizing the translations and ways in which they were resolved were addressed in a separate written report.

**Fig. 1:**
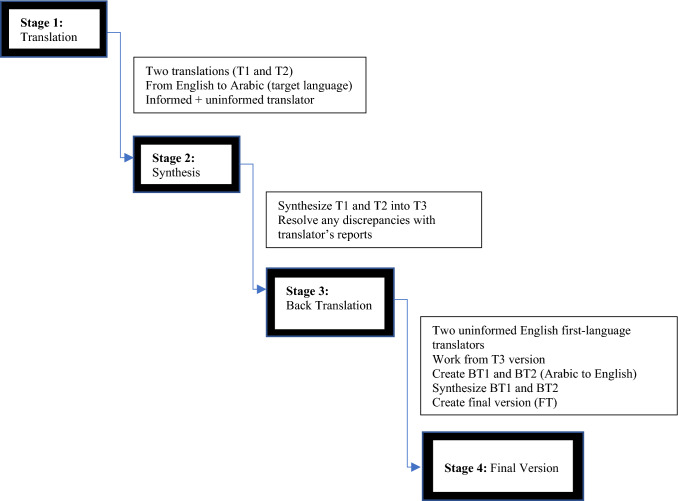
CEFI Translation Methodology (from English to Arabic). Adapted from (Beaton et al., 2000)

The next stage involved two translators (with English as their mother tongue) who are blind to the English version back-translating the CEFI from Arabic to English to check validity (i.e., Arabic and English versions reflect the same item content). They too did not have knowledge of the concepts being quantified nor a developmental or clinical background to avoid biases. The outcomes of this collaboration were two backtranslations (BT1 and BT2). Both translations were then synthesized into the final version (FT). Decisions pertaining to achieving equivalence between the English and Arabic versions in semantic equivalence, idiomatic equivalence, experiential equivalence, and conceptual equivalence (Beaton et al., 2000) were achieved via this translation methodology (see Fig. 1).

All scoring was computerized via the CEFI Scoring Software Program and the MHS Online Assessment Center (https://www.mhsassessments.com) which provides an automated procedure for addressing missing item scores. In addition to EF sub-scales, the CEFI provides standard scores for quality indicators: (a) consistency index (i.e., how consistent / inconsistent the rater’s responses were), (b) positive impression scale (i.e., the extent to which a rater creates an approving impression of the child), (c) negative impression scale (i.e., the extent to which a rater creates an unapproving impression of the child).

### Procedure

Participant recruitment was facilitated via the support of 22 organizations (e.g., autism centers, mainstream schools with inclusion programs for autistic children), across three Emirates (Abu Dhabi, Dubai, Sharjah) from March 2018 to April 2019. Recruitment calls were dispatched through participating schools and centers as well as through research mailing lists, social media groups, and autism-related conferences and workshops. Participants were seen one by one during a research session where language screening assessments were administered. Language status (bilingual or monolingual) was determined following the completion of the research session, upon scoring ratings from direct and parent language measures. CEFI Parent and Teacher forms were administered and collected from families (filled out by either mothers or fathers) and teachers (either head teachers or assigned therapists or shadow teachers at mainstream schools and centers) during home, school or center visits. Raters were administered forms in their language preference (English or Arabic).

### Analysis Methods

Statistical analyses were performed in SPSS Version 24. The data were all found to be normally distributed. Across continuous outcome variables, two-way analysis of variance (ANOVA) were conducted with diagnostic group (Autistic, TD) and language group (monolingual, bilingual) as between subject factors. No co-variate adjustments nor further quality indicators (i.e., positive impression and negative impression scale thresholds) were included in this analyses due to power considerations. Higher standard scores on the CEFI indicate better EF abilities.

## Results

Tables 2 and 3 show the mean standard scores and standard deviations as per parent and teacher EF ratings for children with a consistency index standard score > 75.

**Table 2:** Means and standard deviations from parent EF measures by group

	Monolingual		Bilingual		Range
	Autistic M (SD)	TD M (SD)	Autistic M (SD)	TD M (SD)	
PR: Flexible Switching^B,C^	(n = 21) 87.29 (16.04)	(n = 29) 109.00 (13.51)	(n = 6) 106.83 (17.08)	(n = 24) 104.25 (13.90)	57–135
PR: Interference Control^A,B,C^	(n = 21) 79.33 (14.60)	(n = 29) 106.59 (10.57)	(n = 6) 105.83 (10.81)	(n = 24) 102.67 (11.54)	50–127
PR: Sustained Attention^A,B,C^	(n = 21) 86.43 (13.78)	(n = 29) 105.48 (12.83)	(n = 6) 101.67 (16.81)	(n = 24) 104.96 (11.69)	63–134
PR: Working Memory^B,C^	(n = 21) 85.57 (19.21)	(n = 29) 107.10 (13.86)	(n = 6) 104.33 (14.73)	(n = 24) 102.57 (14.61)	57–137

*M* mean, *SD* standard deviation, *TD* typically developing, *PR* parent rating

^A^Diagnostic effect

^B^Language effect

^C^Interaction effect

**Table 3:** Means and Standard Deviations from Teacher EF Measures by Group

	Monolingual		Bilingual		Range
	Autistic M (SD)	TD M (SD)	Autistic M (SD)	TD M (SD)	
TR: Flexible Switching ^**A**^	(n = 13) 81.23 (12.43)	(n = 8) 113.75 (13.83)	(n = 9) 85.00 (5.91)	(n = 7) 108.71 (16.28)	60–129
TR: Interference Control ^**A**^	(n = 13) 80.23 (16.05)	(n = 8) 111.00 (15.87)	(n = 9) 82.22 (10.12)	(n = 7) 106.29 (15.35)	55–129
TR: Sustained Attention ^**A**^	(n = 13) 83.46 (14.14)	(n = 8) 112.63 (15.57)	(n = 9) 88.00 (9.05)	(n = 7) 105.29 (13.85)	64–124
TR: Working Memory ^**A**^	(n = 13) 82.54 (12.24)	(n = 8) 107.88 (14.50)	(n = 9) 83.44 (4.41)	(n = 7) 105.86 (12.14)	55–119

*M* mean, *SD* standard deviation, *TD* typically developing, *TR* teacher rating

^A^Diagnostic effect

### Group Differences on EF Outcomes

#### Flexible Switching (Parent)

A 2 (diagnostic group) × 2 (language group) ANOVA on parent-rated flexible switching revealed that the main effect of diagnostic group was significant, *F*(1, 76) = 5.92, *p* = 0.017, η_p_^2^ = 0.07 where TD participants displayed significantly better flexible switching than the autistic participants. The main effect of language group was not significant, *F*(1, 76) = 3.54, *p* = 0.064, η_p_^2^ = 0.04, however, the interaction between diagnostic group and language group was significant, *F*(1, 76) = 9.55, *p* = 0.003, η_p_^2^ = 0.11. Post-hoc independent samples t-tests revealed autistic bilinguals had significantly better flexible switching than autistic monolinguals, *t*(25) =  − 2.59, *p* = 0.015.

#### Flexible Switching (Teacher)

A 2 (diagnostic group) × 2 (language group) ANOVA on teacher-rated flexible switching revealed a significant main effect of diagnostic group, *F*(1, 33) = 26.45, *p* = 0.000, η_p_^2^ = 0.44 where the TD participants displayed significantly better flexible switching than the autistic participants. However, neither the main effect of language group, *F*(1, 33) = 0.09, *p* = 0.758, η_p_^2^ = 0.00, nor the interaction effect between diagnostic group and language group, *F*(1, 33) = 1.73, *p* = 0.198, η_p_^2^ = 0.05, were significant.

#### Sustained Attention (Parent)

A 2 (diagnostic group) × 2 (language group) ANOVA on parent-rated sustained attention revealed a significant main effect of diagnostic group, *F*(1, 76) = 10.07, *p* = 0.002, η_p_^2^ = 0.11 where TD participants displayed significantly better sustained attention than the autistic participants. There was a significant main effect of language group, *F*(1, 76) = 4.36, *p* = 0.04, η_p_^2^ = 0.05 where bilinguals exhibited significantly better sustained attention than monolinguals. The interaction effect between language group and diagnostic group was also significant, *F*(1, 76) = 5.01, *p* = 0.02, η_p_^2^ = 0.06. Post-hoc independent samples t-tests revealed autistic bilinguals showed significantly better sustained attention than autistic monolinguals, *t*(25) =  − 2.28, *p* = 0.031.

#### Sustained Attention (Teacher)

A 2 (diagnostic group) × 2 (language group) ANOVA on teacher-rated sustained attention revealed a significant main effect of diagnostic group, *F*(1, 33) = 26.45, *p* = 0.000, η_p_^2^ = 0.44 where TD participants displayed significantly better sustained attention than the autistic participants. There was no significant main effect of language group, *F*(1, 33) = 0.09, *p* = 0.758, η_p_^2^ = 0.00, nor a significant interaction between language group and diagnostic group, *F*(1, 33) = 1.73, *p* = 0.198, η_p_^2^ = 0.05.

#### Interference Control (Parent)

A 2 (diagnostic group) × 2 (language group) ANOVA on parent-rater interference control demonstrated a significant main effect of diagnostic group, *F*(1, 76) = 13.74, *p* = 0.000, η_p_^2^ = 0.15, where TD participants displayed significantly better interference control than the autistic participants. There was a significant main effect of language group, *F*(1, 76) = 12.07, *p* = 0.001, η_p_^2^ = 0.13, where bilinguals exhibited significantly better interference control than monolinguals. The interaction effect between language group and diagnostic group was significant, *F*(1, 76) = 21.91, *p* = 0.000, η_p_^2^ = 0.22. Post-hoc independent samples t-tests revealed autistic bilinguals showed significantly better sustained attention than autistic monolinguals, *t*(25) =  − 4.10, *p* = 0.000.

#### Interference Control (Teacher)

A 2 (diagnostic group) × 2 (language group) ANOVA on teacher-rated interference control revealed a significant main effect of diagnostic group, *F*(1, 33) = 30.68, *p* = 0.000, η_p_^2^ = 0.48, where TD participants displayed significantly better interference control than the autistic participants. However, there was no significant main effect of language group, *F*(1, 33) = 0.07, *p* = 0.785, η_p_^2^ = 0.00, nor an interaction effect between language group and diagnostic group, *F*(1, 33) = 0.45, *p* = 0.503, η_p_^2^ = 0.01.

#### Working Memory (Parent)

A 2 (diagnostic group) × 2 (language group) ANOVA on parent-rated working memory demonstrated a significant main effect of diagnostic group, *F*(1, 76) = 5.16, *p* = 0.026, η_p_^2^ = 0.64, where TD participants displayed significantly better working memory than the autistic participants. There was no significant main effect of language group, *F*(1, 76) = 2.62, *p* = 0.109, η_p_^2^ = 0.03, however, the interaction effect between language group and diagnostic group was significant, *F*(1, 76) = 7.79, *p* = 0.007, η_p_^2^ = 0.09. Post-hoc independent samples t-tests revealed autistic bilinguals exhibited better working memory than autistic monolinguals, *t*(25) =  − 2.20, *p* = 0.037.

#### Working Memory (Teacher)

A 2 (diagnostic group) × 2 (language group) ANOVA on teacher-rated working memory demonstrated a significant main effect of diagnostic group, *F*(1, 33) = 38.26, *p* = 0.000, η_p_^2^ = 0.53, where TD participants displayed significantly better working memory than the autistic participants (see Fig. 1). However, there was no significant main effect of language group, *F*(1, 33) = 0.02, *p* = 0.886, η_p_^2^ = 0.00, nor interaction between language group and diagnostic group, *F*(1, 33) = 0.14, *p* = 0.707, η_p_^2^ = 0.00.

### Relationship Between Parent and Teacher EF Scores

Only 20 participants had both parent and teacher EF data that met the consistency index quality threshold of > 75. Therefore, given the small sample size, the correlational analyses was run with the whole sample of participants who had parent and teacher EF data (including those with lower consistency index scores) which amounts to 55 participants. Therefore, a Pearson’s *r* data analysis on the whole sample (n = 55) revealed strong positive correlations on the following outcome measures, namely: parent and teacher ratings of sustained attention, *r* = 0.63, *p* = 0.000; parent and teacher ratings of flexible switching, *r* = 0.44, *p* = 0.001; parent and teacher ratings of interference control, *r* = 0.72, *p* = 0.000; and parent and teacher ratings of working memory *r* = 0.40, *p* = 0.002.

### Comparing EF Performance Across Raters

Paired samples t-tests on the whole sample (n = 55) revealed no significant differences between parents and teachers on any EF outcome measures: parent sustained attention (*M* = 92.44, *SD* = 15.45) and teacher sustained attention (*M* = 93.15, *SD* = 17.20), *t*(53) =  − 0.36, *p* = 0.715; parent flexible switching (*M* = 94.24, *SD* = 16.88) and teacher flexible switching (*M* = 93.20, *SD* = 17.30), *t*(54) = 0.42, *p* = 0.671; parent interference control (*M* = 87.75, *SD* = 19.29) and teacher interference control (*M* = 89.07, *SD* = 19.36), *t*(54) =  − 0.68, *p* = 0.496; parent working memory (*M* = 94.53, *SD* = 18.73) and teacher working memory (*M* = 90.51, *SD* = 17.57), *t*(54) = 1.50, *p* = 0.139.

## Discussion

The current study investigated the impact of bilingualism in autistic and typically developing children, on a specific set of everyday EF skills measured with parent and teacher reports, in a dual-language environment. All the data were collected in the United Arab Emirates. To our knowledge, this is the first investigation at the interface of bilingualism and autism to use both parent and teacher informant-report measures of EF, and in a group of Arabic-speaking children. The study thus contributes to the diversification of autism research samples; a pressing global issue, in light of the large majority of psychological research that is focused on WEIRD (western, educated, industrial, rich, and democratic) samples (Henrich et al., 2010; Nielsen et al., 2017).

The adaptive control hypothesis suggests that interference control, flexible switching, and sustained attention should all be enhanced for bilinguals, especially for those situated in a dual-language context like ours. We also investigated working memory, as a control domain not hypothesized to be impacted by bilingualism. For parent-reports, consistent interaction effects were noted, indicating a bilingual advantage for autistic participants for all four EF abilities relative to monolingual autistic peers. This finding contrasts with previous studies that used parent-reported EF measures and reported no autistic bilingual advantage (Gonzalez-Barrero & Nadig, 2017; Iarocci et al., 2017) in children. However, this effect of bilingualism was not apparent in teacher-reported EF abilities in the same domains, measured using the same tool—which is consistent with previous studies that employed informant-report EF measures (i.e., parent-reports) for autistic bilingual children (Gonzalez-Barrero & Nadig, 2017; Iarocci et al., 2017).

The finding of widespread bilingual advantage for autistic children across four EF domains should be taken as preliminary evidence and interpreted with caution given the discrepancy of findings between parent and teacher reports. We propose two possible explanations for this discrepancy. First, school and home contexts could encompass different executive demands, resulting in varying perceptions of ability from teachers and parents. Second, our autistic bilingual group is smaller than the other three groups in our study. Small sample size can contribute to greater variability in performance and drive a random effect. However, the consistency of findings within the rater category (all parent-ratings showing an effect of bilingualism for autistic children) argues against this being the relevant explanation.

We also mark the lack of bilingual advantage for TD children across all outcome variables from both CEFI parent and teacher ratings. Since autistic children generally struggle with executive function tasks like those in our study, this might make it easier to detect bilingual advantages in an autistic than a TD sample*.* Nonetheless, our bilingual autistic participants were mostly cognitively abled, school-aged, proficient, living in dual-language contexts and so replications and extensions are required to determine the generalizability of our results.

The consistent finding across parent and teacher reports was a diagnostic effect across most EF outcomes. This indicates autistic children had significantly lower standard scores (poorer EF abilities) relative to TD participants. The presence of this pattern indicates that despite the modest sample size, we did have adequate power to detect diagnostic effects across both EF measures and outcomes. When comparing children’s EF performance across raters (full sample), strong correlations were found between parent and teacher reports across all EF outcomes, as well as a lack of significant differences between parent and teacher raters across all EF outcomes.

There are some limitations worth highlighting. First, despite extensive nation-wide efforts to maximize the study’s sample size (participant recruitment was supported by 20 + institutions across the UAE), we acknowledge that our bilingual autistic sample is under-sized relative to the other three groups. To address a potential loss in power, we carried out Maxwell and Delaney’s (2003) approach to use Type III sums of squares which is resilient to variable group sizes that are subject to comparisons. Second, despite selecting primary caregivers (in the home and school—to fill out parent and teacher reports respectively) who have had extensive and extended quality interactions with our participants, we acknowledge our findings are subject to potential bias in performance, introduced by the informant-report nature of our EF measure.

Third, with regards to the task itself, one could argue that certain CEFI items were not applicable to the children on the younger end of the sample spectrum. An example of this is requesting a parent and/or teacher to rate how well a 5-year old child ‘manages money’ or ‘concentrates while reading’. Milestones relating to money management and reading are rarely achieved in this age group. Similarly, some items are not applicable to raters. An example of this is requesting a parent/teacher to rate whether a child ‘has good thoughts about everyone’. The statement addresses a child’s thoughts, not actions, which can prove difficult for raters to judge. Raters often left items of this nature as unscored or scored as ‘never’, thus introducing a potential bias.

Finally, we cannot be sure to what extent IQ differences might have played a role in performance between autistic bilinguals and monolinguals. However, impaired performance on frontal executive tasks is not fully explained by fluid intelligence (e.g., Roca et al., 2010) and correlations between IQ tests and EFs are not always found in children or adolescents (e.g., Ardila et al., 2000). Friedman et al. (2006) have argued that measures of intelligence do not equally assess the broad range of executive functions. Moreover, age-related differences found in everyday EF, as measured using the BRIEF, in a large group of autistic children remained when covarying out IQ (Rosenthal et al., 2013). Based on some of these previous findings, we are confident that our findings are not solely down to IQ differences across our groups. We acknowledge that ultimately we cannot be sure of this but we are simply underpowered to investigate IQ as a covariate. Needless to say, we believe our study still adds significant value to the small EF evidence-base surrounding autism and bilingualism through the diversification of autism research beyond white and middle-class samples, the first-time inclusion of multiple raters (i.e. parents and teachers), and the first targeted investigation of a prominent theoretical model of bilingualism and EF in an autistic sample.

In terms of strengths and unique contributions, this work makes a valuable contribution to applicability by including autistic children with below average NVIQ—given that all published studies at this interface have only recruited more cognitively-abled autistic participants with average or above average IQ. Furthermore, as far as the raters are concerned, this is the first study at the intersection of bilingualism, autism, and EF to include both parent and teacher perspectives, capturing EF abilities and demands across different life contexts (i.e., home vs. school). Previous studies at this intersection have only included parent raters for informant-type EF investigations.

Using parent ratings, we found an autistic bilingual advantage in EF areas hypothesized to be most impacted by a dual-language context. However, we also found an autistic bilingual advantage in working memory, which is not an EF domain highlighted by the adaptive control hypothesis. It was for this exact purpose that working memory was specifically selected as a control in our study. Therefore, whether our data lends support to the adaptive control hypothesis is unclear. There has only been one prior investigation of this theoretical model at the intersection of bilingualism, autism and EF—a study by Sharaan et al. (2021) that used direct EF assessments. Our findings (based on informant-report assessments) lend partial support to those of Sharaan et al. (2021) in that we did not find support for the ACH, using teacher reports. We also did not find support for the ACH due to the autistic bilingual advantage detected in working memory, an EF domain not predicted by the ACH. On the other hand, we found widespread advantages in the EF domains predicted by the ACH, using parent reports. Therefore, this model would benefit from further testing, which would, in turn, provide supporting or refuting evidence for this theory, thereby also shedding light on the currently debated mechanisms by which cognitive control may be improved in bilinguals.

Our data make it very clear, however, that bilingualism does not result in any EF disadvantages for autistic children, in a dual-language context. Despite concerns from parents and practitioners, we found no evidence that autistic children’s executive function abilities are detrimentally affected by learning and using two languages, in a dual-language context. Together, these findings join a growing body of literature showing that bilingualism does not negatively impact the executive functions of autistic children in a dual-language context, and in fact, might mitigate everyday EF difficulties that they face.

## Supplementary Information

Below is the link to the electronic supplementary material.

Supplementary file1 (DOCX 29 kb)
