# Supplementary material for: The Impact of Bilingualism on Everyday Executive Functions of English-Arabic Autistic Children: Through a Parent-Teacher Lens
Source: J Autism Dev Disord. 2021 Jun 6;52(5):2224–35. doi: 10.1007/s10803-021-05114-5 (PMC9021051; doi:10.1007/s10803-021-05114-5)
Supplement: Supplementary file 1 — Supplementary file1 (DOCX 29 kb) [file 10803_2021_5114_MOESM1_ESM.docx]

**Supplementary Material**

| **Table 4** Means and Standard Deviations from Parent EF Measures by Group: Full Sample Size   \| Monolingual Bilingual \| \| \| \| \|  \|  \| \| --- \| --- \| --- \| --- \| --- \| --- \| --- \| \|  \| Autistic  M (SD) \| TD  M (SD) \| Autistic  M (SD) \| TD Range  M (SD) \|  \|  \| \| PR: Flexible Switching **^A^** \| (n = 32)  87.94 (14.27) \| (n = 32)  108.03 (14.09) \| (n = 18)  93.89 (17.18) \| (n = 35) 57-138  103.97 (14.95) \|  \|  \| \| PR: Interference Control **^A,C^** \| (n = 32)  80.81 (14.67) \| (n = 32)  106.25 (11.95) \| (n = 18)  86.39 (20.85) \| (n = 35) 50-127  100.43 (12.60) \|  \|  \| \| PR: Sustained Attention **^A^** \| (n = 32)  87.22 (12.91) \| (n = 32)  105.91 (12.64) \| (n = 18)  91.22 (15.66) \| (n = 33) 63-134  102.88 (12.29) \|  \|  \| \| PR: Working Memory **^A,C^** \| (n = 32)  87.94 (18.42) \| (n = 32)  106.94 (14.01) \| (n = 18)  96.11 (15.43) \| (n = 33) 57-137  102.29 (14.68) \|  \|  \| \|  \|  \|  \|  \|  \|  \|  \|   *Note.* M = mean; SD = standard deviation; TD = typically developing; PR = parent rating; ^A^ = diagnostic effect; ^B^ = language effect; ^C^ = interaction effect. |
| --- | --- | --- | --- | --- | --- | --- | --- | --- | --- | --- | --- | --- | --- | --- | --- | --- | --- | --- | --- | --- | --- | --- | --- | --- | --- | --- | --- | --- | --- | --- | --- | --- | --- | --- | --- | --- | --- | --- | --- | --- | --- | --- | --- | --- | --- | --- | --- | --- | --- |

**Parent-Reported Group Differences on EF Outcomes Using Full Sample Size**

*Flexible Switching (Parent)*

A 2 (diagnostic group) x 2 (language group) ANOVA on parent-rated flexible switching revealed that the main effect of diagnostic group was significant, *F*(1, 113) = 27.96, *p* = .000, η_p_^2^ = .19 where TD participants displayed significantly better flexible switching than the autistic participants. The main effect of language group was not significant, *F*(1, 113) = 0.11, *p* = .741, η_p_^2^ = .00. Similarly, the interaction between diagnostic group and language group was not significant, *F*(1, 113) = 3.07, *p* = .082, η_p_^2^ = .02.

*Sustained Attention (Parent)*

A 2 (diagnostic group) x 2 (language group) ANOVA on parent-rated sustained attention revealed that the main effect of diagnostic group was significant, *F*(1, 111) = 36.00, *p* = .000, η_p_^2^ = .24 where TD participants displayed significantly better flexible switching than the autistic participants. The main effect of language group was not significant, *F*(1, 111) = 0.37, *p* = .847, η_p_^2^ = .00. Similarly, the interaction between diagnostic group and language group was not significant, *F*(1, 111) = 1.93, *p* = .167, η_p_^2^ = .01.

*Interference Control (Parent)*

A 2 (diagnostic group) x 2 (language group) ANOVA on parent-rater interference control demonstrated a significant main effect of diagnostic group, *F*(1, 113) = 50.25, *p* = .000, η_p_^2^ = .30, where TD participants displayed significantly better interference control than the autistic participants. There was no significant main effect of language group, *F*(1, 113) = 0.00, *p* = .965, η_p_^2^ = .00. There was, however, a significant interaction effect between language group and diagnostic group, *F*(1, 113) = 4.18, *p* = .043, η_p_^2^ = .03. Post-hoc independent samples t-tests revealed autistic bilinguals showed significantly better interference control than autistic monolinguals, *t*(48) = -1.10, *p* = .034.

*Working Memory (Parent)*

A 2 (diagnostic group) x 2 (language group) ANOVA on parent-rated working memory demonstrated a significant main effect of diagnostic group, *F*(1, 113) = 17.45, *p* = .000, η_p_^2^ = .13, where TD participants displayed significantly better working memory than the autistic participants. There was no significant main effect of language group, *F*(1, 113) = 0.34, *p* = .560, η_p_^2^ = .00, however, the interaction effect between language group and diagnostic group was significant, *F*(1, 113) = 4.52, *p* = .035, η_p_^2^ = .03. Post-hoc independent samples t-tests revealed autistic bilinguals exhibited better working memory than autistic monolinguals, *t*(48) = -1.59, *p* = .042.
